# AI-Enabled Reduction of Animal Use in Cardiovascular Translational Medicine: Regulatory and Technological Perspectives

**DOI:** 10.3390/life15121916

**Published:** 2025-12-14

**Authors:** Rasit Dinc, Nurittin Ardic

**Affiliations:** 1 INVAMED Medical Innovation Institute, One World Trade Center, 85th Floor, 285 Fulton Street, New York, NY 10007, USA; 2 Med-International UK Health Agency Ltd., Leicestershire LE10 0BZ, UK; nurittinardic@yahoo.com

**Keywords:** artificial intelligence, New Approach Methodologies (NAMs), cardiovascular safety, digital twins, regulatory science, in vitro–in silico translation

## Abstract

Background: Animal studies remain fundamental to cardiovascular drug and device development, yet their ability to predict human responses is increasingly being questioned. The US Food and Drug Administration (FDA)’s April 2025 roadmap supports alternative testing approaches that strategically reduce animal use while increasing human relevance by combining laboratory methods, computer simulations, and artificial intelligence. This review examines AI-enabled alternative methodologies for cardiovascular safety assessment within established validation frameworks and regulatory acceptance programs. We describe machine learning approaches for predicting cardiac safety risks, automated analysis of human heart cells, and patient-specific computer simulations for evaluating medical devices. These tools can improve our understanding of biological mechanisms, focus limited animal studies on critical questions, and accelerate decision-making. Regulatory acceptance requires rigorous validation appropriate to each specific use and decision context. Conclusion: We outline practical steps for establishing credibility, including transparent data documentation, independent testing, and identifying where models can be reliably applied, and identify remaining challenges in data standardization and regulatory readiness. With ongoing alignment between regulators, standards bodies, and product developers, these alternative approaches could significantly reduce reliance on animal testing in cardiovascular research while maintaining or improving the quality of evidence.

## 1. Introduction

Cardiovascular diseases (CVDs) remain the leading cause of death. While traditional pharmacotherapy and surgery can alleviate symptoms and reduce mortality, effective clinical strategies to prevent serious outcomes remain lacking. Animal experiments have historically formed the foundation not only of other medical fields but also of cardiovascular translational research, informing pharmacology, device design, and toxicity assessment. However, interspecies differences in ion channels, heart rate, metabolism, and inflammatory responses create fundamental limitations [1,2]. The cardiovascular field is uniquely positioned to benefit from the transition to AI-powered NAMs. First, animal models show particularly poor predictive accuracy for key cardiovascular safety endpoints: hERG channel blockade, QT prolongation, cytokine-mediated inflammation, and thrombogenicity often manifest differently across species [1,2,3]. Second, the field has access to a wealth of human-relevant alternatives: induced pluripotent stem cell-derived cardiomyocytes (iPSC-CMs) that mimic human electrophysiology, organ-on-a-chip platforms that model vascular biology, and comprehensive clinical registries that provide real-world validation data. Third, computational cardiovascular modeling has matured significantly, and physics-based simulations of cardiac electrophysiology and hemodynamics are now capable of making patient-specific predictions when combined with machine learning acceleration. Traditional rodent or primate studies often fail to capture adverse reactions that occur only in humans, such as arrhythmic predispositions or cytokine release syndrome [3].

While previous reviews have addressed the regulatory frameworks or technical capabilities of alternative methods separately, this review offers the first integrated analysis following the FDA’s transformative 2025 roadmap. We synthesize regulatory requirements, validation frameworks, and practical implementation guidance to provide developers with actionable pathways for adopting AI-powered alternative methods. Our unique contribution lies in linking regulatory policy (FDA 2025 roadmap, international harmonization efforts) to technical validation standards (ASME V&V 40, OECD guidelines) and real-world implementation barriers (economic viability, institutional capacity, technology accessibility). This integrated perspective addresses a critical gap: Knowing “what” alternative methods exist is not enough without understanding “how” to validate them for regulatory acceptance and whether they are practically “applicable” to different types of organizations.

Legal and regulatory changes are now enabling this transition. In late 2022, The United States Congress passed FDA Modernization Act 2.0, which explicitly allows the use of non-animal alternatives (cell-based assays, computer models, etc.) to support an investigational new drug (IND) application and “removes the requirement to use animal studies” for biosimilar biologics license applications (BLAs) [4]. The FDA’s requirement for animal testing will be reduced, improved, or potentially replaced using a number of approaches, including AI-based toxicity computational models and in vitro organoid toxicity testing with cell lines (called New Approach Methodologies or NAM data) [5].

Building on this foundation, the Food Drug Administration (FDA)’s 2025 Animal Experimentation Reduction Roadmap outlines practical pathways for integrating NAMs (advanced in vitro systems, microphysiological platforms, and AI-powered computational models) into regulatory submissions [5,6]. The roadmap sets a 3–5-year horizon to make animal testing the exception rather than the rule, with initial pilot studies focusing on monoclonal antibodies (mAbs) before expanding to small molecules, biologics, and medical devices [7]. To make efficacy determinations, the agency will also begin using pre-existing, real-world safety data from other countries with comparable regulatory standards where the drug has previously been studied in humans [5]. Similar initiatives from the European Medicines Agency (EMA), Organisation for Economic Co-operation and Development (OECD), and National Institutes of Health (NIH) converge on a common goal: scientific credibility and ethical mitigation [8,9].

**Figure 1:**
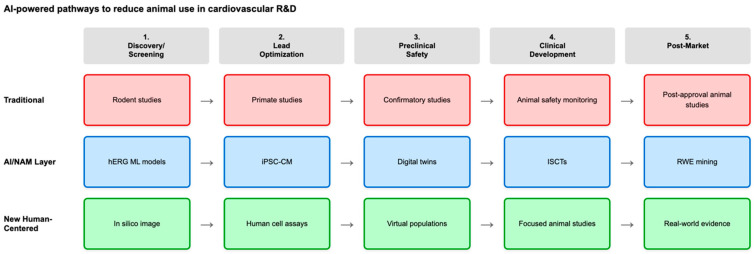
Schematic representation of the phased integration of AI-enabled novel approach methodologies (NAMs) and human-centered models throughout the cardiovascular R&D process. The cardiovascular drug and device development process has traditionally relied heavily on animal studies at every stage (top path, red). The AI-enabled NAM transformation (middle path, blue) offers computational models, and real-world evidence (RWE) that could replace or significantly reduce animal use at every stage. The bottom path (green) row represents human-centered approaches that emphasize in silico and human-based evidence and focus the remaining animal studies on confirmatory safety endpoints.

The new approach is designed to improve drug safety and accelerate the evaluation process, while also reducing animal testing, research and development (R&D) costs, and ultimately, drug prices. Artificial intelligence is supporting this shift by enabling predictive toxicology, automated pattern recognition, and large-scale human-specific simulation. Machine learning (ML) models for cardiac ion channel risk, automated phenotyping of iPSCs)-CMs, and patient-specific digital twins together form a new translational ecosystem (Figure 1).

In cardiovascular contexts, where precise prediction of electrophysiology, hemodynamics, and thrombosis is crucial, the need for more reliable models is paramount. This review examines the regulatory framework, technical foundations, validation requirements, and future directions for AI-enabled NAMs in cardiovascular research.

This narrative review synthesizes current knowledge on alternative AI-enabled methodologies for cardiovascular research, highlighting regulatory and practical perspectives. Our analysis draws on systematic literature searches in PubMed, Scopus, and Google Scholar (2018–2025), prioritizing peer-reviewed research, regulatory guidance documents (FDA, EMA, OECD), and validation reports from standards organizations. We organize our findings thematically, moving from policy foundations to technical capabilities and practical implementation.

To our knowledge, this is the first review to combine regulatory frameworks (FDA, EMA, OECD), AI-assisted alternative methods, and cardiovascular-specific applications in a single, comprehensive analysis covering discovery, preclinical evaluation, and clinical translation. Section 2 examines the evolving regulatory landscape, including the FDA 2025 roadmap and international perspectives. Section 3 examines current AI technologies organized by drug development stage (discovery, preclinical, clinical application). Section 4 examines digital twins, in silico clinical trials, and real-world evidence from the clinical implementation phase. Section 5 provides a critical analysis of validation requirements and current limitations. Section 6 addresses ethical and economic implications and practical barriers. Section 7 outlines future directions with realistic timelines.

## 2. Regulatory Environment and 2025 FDA Roadmap

### 2.1. Policy Evolution

The FDA Animal Experimentation Reduction Roadmap (April 2025) represents a paradigm shift in preclinical safety assessment [5,6]. The Roadmap expands the Alternative Methods Development (AAMs) program and prioritizes: (i) ensuring the qualification of NAMs for regulatory submissions, (ii) expanding AI-powered predictive models, and (iii) cross-sector harmonization through the Interagency Coordinating Committee for the Validation of Alternative Methods (ICCVAM) [10]. The agency emphasizes quantitative validation that is “credible within the intended scope” rather than universal animal benchmarking. Furthermore, it recognizes that human-relevant methods may outperform animal models in predicting human outcomes [1,2].

The roadmap begins with monoclonal antibodies as a test case due to extensive regulatory requirements and poor animal–human compatibility [6,7]. Current FDA requirements for monoclonal antibodies (mAbs) mandate Good Laboratory Practice (GLP)-compliant repeat-dose toxicity studies (typically 1–6 months in duration) in non-human primates, costing often estimated over $2 million per study [11,12]. However, immunogenicity in animals often confounds toxicity interpretation, and some safety risks (e.g., cytokine release syndrome) are not detected in animal studies [13]. The Roadmap recommends shortening routine 6-month primate toxicology studies to 3 months if 1-month studies and NAM tests show no alarming signals, potentially reducing study duration and associated animal use [7,13].

### 2.2. Standards and Global Harmonization

The FDA’s Computational Modeling and Simulation (CM&S) guideline (2023) and the recognition of the American Society for Medical Engineers Verification and Validation (ASME V&V) 40-2018 establish the credibility framework for computational evidence in medical device applications [14,15]. For devices, CM&S can now replace certain benchtop or animal studies if verification, validation, and uncertainty quantification (VVUQ) are demonstrated by evidence proportional to the decision risk [14]. This risk-based approach clearly links the required level of credibility evidence to the regulatory question being addressed.

The OECD Guidance Document on Good In Vitro Method Practices (GIVIMP, 2018) and the EMA Regulatory Science to 2025 strategy similarly encourage harmonized validation of NAMs across jurisdictions [8,9]. Together, these create a globally consistent regulatory environment where validated AI-based NAMs can support international applications and reduce duplicative animal testing across regions.

### 2.3. Regulatory Considerations Specific to Cardiovascular Products

Cardiovascular device and drug developers can directly benefit from these frameworks. The FDA’s Medical Device Development Toolkit (MDDT) program has begun to include computational tools [16]. The Innovative Science and Technology Approaches for New Drugs (ISTAND) is an FDA program to qualify new drug development tools (DDTs) that fall outside existing regulatory pathways. By emphasizing context-of-use (CoU) and risk-based evidence requirements, the FDA’s ISTAND and Predictive Toxicology Roadmap programs assess and advance the use of NAMs, alternatives to animal testing. These roadmaps aim to accelerate the adoption of innovative technologies by building confidence in new scientific approaches [17]. For cardiovascular applications, this means that AI models for cardiotoxicity prediction, digital twins for device design optimization, and organ-on-a-chip systems for thrombogenicity assessment could serve as primary or supporting evidence, depending on the rigor of validation.

### 2.4. Ethical and Translational Implications

**Figure 2:**
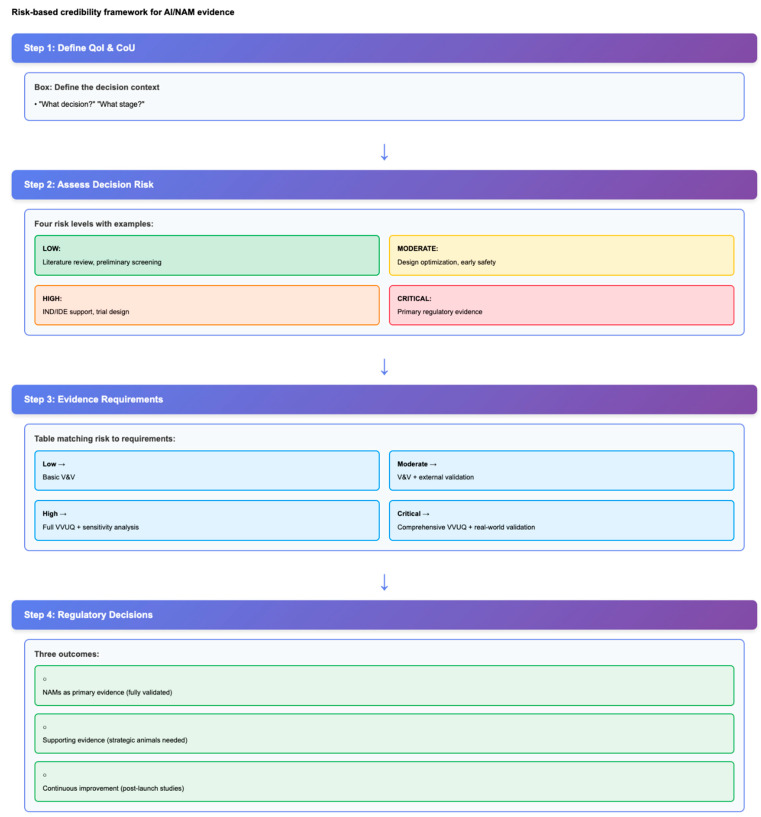
Risk-based reliability framework for AI/NAM evidence. FDA’s risk-informed reliability assessment framework for computational models and AI tools follows a four-step process. Decision tree: Step 1 defines the Question of Interest (QoI) and Context of Use (CoU) to determine which decision the model will inform and at which regulatory stage. Step 2 assesses decision risk at four levels (Low/Moderate/High/Critical) and identifies relevant cardiovascular application examples at each level. Step 3 specifies the reliability evidence requirements scaled by risk: verification and validation (all levels), validity assessment (medium-high), uncertainty quantification (UQ) (high-critical), and applicability domain documentation (all AI/ML). Step 4 maps reliability evidence to regulatory decisions: NAMs as primary evidence when fully validated, supporting evidence requiring strategic confirmatory animals when gaps exist, and continuous improvement through validation after launch. In Step 2, green denotes low risk, yellow denotes moderate risk, orange denotes high risk, and red denotes critical risk.

The FDA advocates for strategic animal reduction rather than complete elimination. The NAMs and AI are used for discovery, screening, and optimization, while minimal confirmatory animal studies are reserved for critical gaps where human data are lacking [5,6]. This aligns with the 3Rs (Replace, Reduce, Refine) and adds a “fourth R”: reliability. By increasing human relevance and reducing interspecies variability, NAMs promise better predictability as well as ethical progress. The FDA’s risk-based credibility framework (Figure 2) aligns evidence certainty with decision risk, enabling flexible AI/NAM integration while maintaining appropriate regulatory standards.

### 2.5. International Perspectives and Global Harmonization

While the FDA’s 2025 roadmap provides leadership, successful adoption of alternative methods requires global coordination. Regulatory requirements and approval processes vary significantly across jurisdictions, creating both challenges and opportunities for cardiovascular product developers.

China (NMPA): The National Medical Products Administration has published specific technical guidelines for AI medical devices, including principles for the classification of AI-based medical software and related registration requirements [18]. China’s approach emphasizes clinical validation within Chinese populations and requires local data for approval even when international evidence is available. For cardiovascular devices that incorporate computational modeling, the NMPA requires validation against Chinese patient anatomical databases. The regulatory timeline for new AI applications typically extends by 18–24 months, and acceptance of alternative methods is slower than traditional animal studies. However, recent initiatives under the 14th Five-Year Plan prioritize reducing animal testing in line with international standards [19].

Japan (PMDA): The Medicines and Medical Devices Agency (MHRA), while applying conservative requirements for computational evidence, has designated specific pathways for innovative technologies through the SAKIGAKE designation system [20]. For cardiovascular medical devices, the PMDA accepts computational modeling as supporting evidence, but rarely as primary evidence, and generally requires confirmatory animal studies even when robust simulations are available. Japanese regulatory culture emphasizes comprehensive pre-application consultation (sōdan), which allows developers to agree on alternative testing strategies on a case-by-case basis [21]. A recent PMDA-FDA collaboration through the International Medical Device Regulators Forum (IMDRF) aligns computational modeling acceptance criteria [22].

United Kingdom (MHRA): Following Brexit, the Medicines and Healthcare products Regulatory Agency (MHRA) has positioned itself as an innovation-friendly regulator, particularly for AI and alternative methods. The Innovative Licensing and Access Pathway (ILAP) provides expedited pathways for products using novel evidence packages, including those for alternative method-based cardiovascular applications. The MHRA has adopted ASME V&V 40 validation for computational models and has approved several cardiovascular device applications using digital twin simulations as primary evidence. The UK’s regulatory flexibility offers opportunities for early adoption but creates potential challenges for global harmonization [23].

European Union (EMA): The European Medicines Agency’s strategy to 2025 explicitly prioritizes alternative methods, with cardiovascular applications identified as high-priority areas. The Innovation Task Force provides scientific advice on new methodologies, and the latest guidance (EMA/CHMP/ICH 2024) clarifies acceptance criteria for physiologically based pharmacokinetic modeling and quantitative systems pharmacology. However, acceptance during decentralized procedures varies among EU Member States [24].

Harmonization Studies: The International Council for Harmonization (ICH) is developing guidelines (ICH M3-R3) addressing alternative methods in non-clinical safety assessment. The OECD continues to expand its Test Guideline Programme to include computationally derived endpoints. These harmonization efforts aim to reduce duplication of testing across jurisdictions by providing mutual recognition of alternative method validations. Strategic regulatory planning for cardiovascular developers should account for jurisdictional differences while anticipating convergence toward harmonized standards over the next 3–5 years [25].

Table 1 summarizes key regulatory differences across major jurisdictions, highlighting differences in alternative method acceptance, review pathways, and timeline expectations that cardiovascular developers must follow for global product development.

### 2.6. New Ethical Considerations in AI-Enabled Alternative Methods

While alternative methods advance the traditional 3R ethical framework (Replace, Reduce, Improve), they introduce new ethical considerations that must be carefully considered, particularly in cardiovascular applications where prediction errors can have serious consequences.

Algorithmic Bias and Health Equity: Machine learning models trained on historically available datasets often reflect existing healthcare disparities. Cardiovascular AI models developed primarily on European or North American populations may underperform for underrepresented demographic groups. For example, Rajkomar et al. (2018) documented that cardiovascular risk prediction algorithms exhibit differential accuracy across racial groups due to the composition of the training data [26]. Obermeyer et al. (2019) demonstrated systematic bias in algorithms predicting health needs, with Black patients requiring higher disease severity than White patients to receive equivalent risk scores [27]. In the cardiovascular domain, this manifests as potentially inaccurate cardiotoxicity predictions or suboptimal device simulation results for populations underrepresented in the training data.

Addressing biases requires: (1) Diverse and representative training datasets encompassing ethnic, age, gender, and geographic populations; (2) Mandatory subgroup validation reporting documenting model performance across demographics; (3) Continuous monitoring of performance disparities in post-market surveillance; (4) Clear applicability domain definitions that identify populations where predictions remain uncertain.

Transparency and Explainability: Unlike traditional animal studies where biological observations can be directly interpreted, deep learning cardiovascular models often function as “black boxes” that generate predictions without providing mechanistic explanations. This creates challenges for regulatory review and clinical confidence. The FDA’s AI/Machine Learning Action Plan (2023) emphasizes explainable AI requirements, particularly for high-risk medical applications. For cardiovascular alternative methods, this means developers must provide: (1) Feature importance analyses that demonstrate which biological parameters drive the predictions; (2) Sensitivity analyses that demonstrate how parameter changes affect the results; and (3) Mechanistic interpretability that relates computational results to known cardiovascular physiology [15].

Informed Consent and Data Privacy: Large-scale cardiovascular alternative method validation requires comprehensive patient data (electrocardiograms, imaging, clinical outcomes). Unlike animal research, which is managed by institutional animal care committees, the use of human data raises complex consent and privacy issues. Integration of real-world evidence using electronic health records and digital twin validation must balance scientific needs with patient privacy rights under GDPR, HIPAA, and new AI-specific regulations.

Validation Ethics: Alternative methods that inadequately predict human safety risks can paradoxically harm both animals and humans; the continued use of invalid tests in animals and inadequate safety assessment in humans. This creates an ethical imperative for rigorous validation before claiming equivalence or superiority to animal studies. Premature regulatory acceptance of poorly validated alternative methods will undermine both scientific credibility and the 3R framework itself.

The cardiovascular field must proactively address these ethical dimensions through diverse stakeholder engagement, transparent validation reporting, and ongoing monitoring for unanticipated outcomes. Ethical progress requires not only replacing animal testing but also ensuring that alternative methods serve all populations equally and safely.

### 2.7. Patient and Public Perspectives

Public attitudes toward animal testing and alternative methods significantly influence regulatory policy and industry practices. Understanding patient and advocacy perspectives ensures that alternative method development aligns with societal values and maintains public confidence in medical product safety.

Trust and Perception of Safety: Focus groups with cardiovascular patients reveal that trust in alternative methods depends largely on validation transparency and regulatory oversight. Patients express concern that computer simulations or laboratory cell cultures may miss important safety signals identified in all animal studies. These concerns are partially offset when alternative methods are presented as complements, rather than wholesale replacements, for strategic animal testing. Clear communication about validation processes and regulatory review standards enhances patient trust.

Positions of Advocacy Organizations: Major cardiovascular patient advocacy groups (American Heart Association, European Society of Cardiology patient representatives) have issued statements supporting alternative method development while emphasizing the priority of evidence for safety and efficacy. These organizations call for the following: (1) Patient representation in alternative method validation planning; (2) Transparent reporting of validation results, including limitations; (3) Post-market surveillance to identify safety signals potentially missed by alternative methods; (4) Continuous investment in both alternative methods and improving animal model suitability when animal studies are necessary.

Participation Strategies: Meaningful patient input in alternative method development requires structured participation beyond symbolic representation. Best practices include the following: (1) Patient advisory boards participating in validation study design; (2) Plain-language summaries of validation results accessible to non-scientists; (3) Public workshops explaining the operation and limitations of alternative methods; (4) Patient participation in regulatory guideline development (FDA Patient Representative Program). The cardiovascular field has pioneered several patient participation models that provide templates for the adoption of alternative methods (e.g., patient input in left ventricular assist device development).

Balancing Perspectives: Patients and public stakeholders generally support the 3R ethical framework when prioritizing safety assurance. This obligates alternative method developers to demonstrate not only equivalence with animal studies but also superiority in predicting human cardiovascular responses. Successfully balancing these perspectives requires ongoing dialogue, transparency about both successes and failures, and adherence to rigorous validation standards that serve both ethical and scientific goals.

## 3. AI-Enabled Technologies: Current Capabilities by Development Stage

AI and alternative methods span the entire cardiovascular product development continuum, from early discovery to clinical implementation. We organize existing technologies by development stage (discovery and screening, preclinical testing, and clinical implementation) to demonstrate where alternatives can replace, mitigate, or improve animal studies at each decision point. Within each stage, we categorize technologies by regulatory maturity: methods with documented successful regulatory applications; methods with promising but limited regulatory precedent; and proof-of-concept approaches requiring significant additional work.

### 3.1. Discovery and Early Screening Technologies

Early-stage drug development has traditionally relied on high-throughput animal screening for cardiotoxicity risk. AI-powered in vitro and in silico methods now offer faster and more cost-effective alternatives for initial compound triage.

Cardiac Ion Channel Prediction: Deep learning (DL) models predicting human ether-à-go-go-related gene (hERG) potassium blockade represent the most mature alternative method for cardiovascular safety assessment. hERG blockage prolongs cardiac repolarization (QT prolongation, drug-induced QT prolongation), creating a risk of arrhythmias that have caused numerous drug withdrawals. Cai et al. (2019) developed deep neural networks trained on over 9000 compounds with experimental hERG data and achieved a prediction accuracy (area under the receiver operating characteristic curve, AUROC) of 0.89 on retained test sets [28]. Delre et al. (2022) extended this using gradient boosting on integrated drug databases and demonstrated robust performance (AUROC 0.87–0.92) across diverse chemical structures [29].

These models have gained regulatory acceptance: the FDA’s Comprehensive In Vitro Proarrhythmia Testing (CiPA) initiative recognizes in silico hERG prediction as acceptable evidence for early-stage cardiac safety assessment when the models meet defined performance criteria (sensitivity ≥ 80%, specificity ≥ 75% in validation sets) [30]. Many pharmaceutical companies now use hERG prediction models as primary tier 1 screening, screening high-risk compounds prior to synthesis. This replaces early-stage animal testing for thousands of compounds each year while increasing human relevance; in silico predictions of human hERG blockade are better at predicting human QT effects than guinea pig or rabbit animal models (concordance: in silico 85–90%, animal models 60–70%) [31].

Electrocardiogram-Based Risk Detection Beyond in vitro channel prediction, electrocardiogram (ECG)-based convolutional neural networks (CNNs), such as Query-Based Temporal Fusion Network (QTNet), drug-induced QT prolongation in real-world clinical settings. Zhang et al. (2024) developed QTNet, which predicts QT prolongation from single-lead ambulatory ECGs with 84% sensitivity and 78% specificity [32]. Alam et al. (2024) demonstrated similar deep learning architectures for automatic QT monitoring in resource-limited environments using smartphone-acquired ECGs [33].

While these approaches hold promise for clinical safety monitoring, they lack comprehensive regulatory precedents as primary evidence. Current practices focus on supplemental clinical trial safety monitoring rather than replacing preclinical studies. Validation challenges include: (1) performance degradation across different ECG acquisition devices; (2) limited data from drugs in development (most training uses post-market approved drugs); and (3) unclear regulatory standards for accepting algorithmic ECG interpretation as a primary endpoint.

Discovery-stage AI methods have gained the clearest regulatory acceptance, particularly for hERG/QT prediction. These tools have proven humane while reducing early-stage animal use. However, even “regulation-ready” methods require case-by-case evaluation; no model is universally accepted across all chemical domains. Emerging ECG-based approaches, while promising, require additional validation, particularly for prospective application to drugs in development rather than retrospective analysis of marketed drugs. The cardiovascular field offers templates for broader adoption, leading other therapeutic areas in the maturity of alternative discovery-stage methods.

### 3.2. Artificial Intelligence-Enhanced Human Cell and Organ-on-Chip Analyses

High-content imaging of iPSC-derived cardiomyocytes combined with DL captures subtle morphological toxicity patterns that manual analysis misses. Grafton et al. (2021) demonstrated that CNN analyzing cardiomyocyte images detect cardiotoxicity with higher sensitivity than traditional analyses and identify compounds that later become problematic in clinical development [34]. These human-focused analyses provide electrophysiology and morphology endpoints that map more directly to clinical safety signals compared to animal screens.

Automatic monitoring of contractility, heart rate variability, and calcium handling using computer vision (CV) algorithms enables standardized phenotyping across laboratories [35]. The FDA’s NAMs application portal (2025) is actively defining performance criteria for cardiac microphysiological systems to standardize validation and facilitate regulatory acceptance [36]. Human iPSC-derived cardiomyocytes (hiPSC-CMs) and microphysiological systems assays with AI-assisted analysis can replace exploratory animal screens in safety pharmacology and provide mechanistic insights into human-specific toxicity pathways not available from animal models [34,36]. However, interlaboratory protocol variability and lack of consensus validation standards currently limit its regulatory acceptance as primary evidence.

### 3.3. Mechanistic Modeling and Ml Surrogates

Hybrid models combine mechanical simulations of cardiac electrophysiology and hemodynamics with ML surrogates for population-scale uncertainty quantification. Domínguez-Gómez et al. (2024) [37] developed emulators that reproduce full multi-ion channel cardiac models with a >100-fold increase in computational speed. This computationally enables virtual population modeling that is not possible with traditional simulations alone [37]. These emulators support rapid exploration of parameter spaces representing anatomical and physiological variability while preserving mechanistic interpretability.

Sex-specific and population-stratified emulators allow for the assessment of differential drug responses across demographics without requiring separate animal studies for each subgroup [37]. This approach is particularly valuable for cardiovascular applications, where patient heterogeneity significantly impacts outcomes. However, emulator validation requires extensive comparison with mechanistic models and clinical data to ensure that predictions remain physiologically plausible in the parameter space.

### 3.4. PK/PD and Systems Pharmacology Modeling

ML-enhanced physiologically based pharmacokinetics/pharmacodynamics (PBPK/PD) and quantitative systems pharmacology (QSP) frameworks predict exposure-response relationships and organ-level toxicity with reduced dependence on species scaling [38]. Integration with AI-optimized parameter estimation accelerates the translation of complex biologics and small molecule cardiovascular drugs by leveraging human in vitro data and clinical pharmacokinetic information to complement animal pharmacokinetic studies.

For monoclonal antibodies targeting cardiovascular indications (e.g., PCSK9 inhibitors, anti-IL-1β for atherosclerosis), PBPK/QSP modeling leveraging human tissue distribution data represents a promising direction that could reduce or complement, rather than eliminate, long-term primate pharmacokinetic studies [11,12]. Current regulatory guidance requires case-by-case evaluation with computational approaches serving as supporting evidence rather than primary evidence for most biologic applications.

### 3.5. Network Pharmacology Approaches

Graph-based AI models elucidate pathway-level cardiotoxicity by linking molecular targets to clinical phenotypes through biological networks [39]. These data-driven “in silico networks” integrate omics data, drug-target interactions, and adverse event databases to predict cardiovascular safety risks based on mechanism-of-action profiles. Network pharmacology enables mechanistic hypothesis generation without animal experiments, guiding rational design decisions in the early stages of development.

While network pharmacology appears promising for early-stage prioritization, this approach is still in the proof-of-concept phase for regulatory applications. Challenges include incomplete biological network knowledge, difficulty experimentally validating predicted mechanisms, and limited regulatory precedent for network-based evidence. Current practices focus on hypothesis generation and target identification rather than definitive safety assessment. Table 2 summarizes representative AI models and maps each technology based on its cardiovascular application, animal reduction potential, and supporting evidence.

AI-powered alternative methods span the cardiovascular development pipeline with varying levels of regulatory maturity. Discovery-stage applications (hERG/QT prediction) have achieved regulatory acceptance and demonstrable animal reduction. Preclinical-stage technologies (iPSC-CMs with AI analysis, mechanistic modeling) offer strong technical promise but face standardization challenges that limit regulatory precedents. Advanced approaches (PBPK/QSP for biologics, network pharmacology) are in the proof-of-concept phase and require significant additional validation before regulatory acceptance. Critically, even mature methods require case-by-case evaluation within defined applicability domains; no approach achieves general regulatory acceptance. The field is progressing not through complete replacement of animal studies but through strategic reduction: computational and in vitro methods are used for screening and optimization, while focused animal studies are reserved for critical safety questions where alternatives fall short. Success requires a realistic assessment of the current maturity of each technology and the appropriate alignment between the methods and the regulatory decision context.

## 4. Digital Twins, In Silico Experiments, and Real-World Data

### 4.1. Cardiac Digital Twins and Population Modeling

Personalized digital twins (DTs) integrate medical imaging, electrocardiography, and clinical data into physics-constrained computational models that simulate individual patient physiology. Qian et al. (2025) developed a framework for creating population-scale cardiac digital twins supported by multimodal data from over 3000 individuals and disease-specific cohorts in the UK Biobank [40]. These population DTs capture anatomical and electrophysiological variability across demographics, enabling virtual experiments on cardiovascular interventions without animal testing. The computational process includes the following: (1) automatic segmentation of cardiac structures from imaging data using deep learning; (2) generation of patient-specific finite element networks; (3) calibration of biophysical models based on individual physiological measurements; and (4) UQ of margin estimates [40,41]. This approach transforms static imaging into dynamic, personalized simulations suitable for device design evaluation and treatment planning.

### 4.2. In Silico Clinical Trials (ISCTs)

Systematic reviews indicate that ISCTs are increasingly being applied to cardiovascular interventions and have a maturing methodology for validation and reporting. Rodero et al. (2023) reviewed 127 studies using ISCTs for cardiac applications, finding increasing use in arrhythmia management, heart failure treatment optimization, and device design [41]. Studies incorporating ASME V&V 40 validation demonstrate agreement between virtual cohort predictions and clinical registry results, supporting regulatory acceptance [42,43].

For transcatheter aortic valve implantation (TAVI), computational fluid dynamics (CFD) and finite element analysis (FEA) simulations assess valve stress, flow patterns, paravalvular leak risk, and device durability in virtual patient anatomies [44,45]. Catalano et al. (2025) and Scuoppo et al. (2025) systematically UQ by applying the ASME V&V 40 methodology to patient-specific TAVI modeling and validated predictions against clinical endpoints [42,43]. These studies demonstrate that properly validated ISCTs can replace early-stage animal design iterations by focusing the limited number of confirmatory animal studies on specific regulatory questions.

A case study modeling in the US might be as follows: A typical TAVI device development traditionally requires multiple sheep studies for design iterations and chronic durability assessment, and these studies encompass approximately 56 animals over 24 months [44,45]. Sheep studies are considered the gold standard for preclinical durability testing because calcification rates in young sheep correlate with the long-term clinical durability of tissue valves [44]. An ISCT-based approach can reduce both animal use and development timelines by using virtual cohorts (*n* ≈ 2000 digital patients) for design optimization followed by a single confirmatory study (*n* ≈ 12 animals) to address specific regulatory questions [44,45]. Such approaches align with the FDA’s strategic mitigation framework, where in silico modeling focuses animal studies solely on critical gaps not yet adequately addressed by computational methods.

### 4.3. Real-World Evidence (RWE) and Post-Market Ai Surveillance

AI mining of electronic health records (EHRs) and international pharmacovigilance databases provides human-specific safety data that complements or replaces older animal repeated-dose studies [46]. ML models identify patterns of cardiovascular adverse events across diverse populations, enabling risk prediction for new treatments based on mechanism of action similarity to approved drugs [46,47]. The FDA’s 2025 Roadmap specifically mentions integrating real-world data from countries with comparable regulatory standards to validate NAM performance and inform regulatory decisions [5,6].

Natural language processing (NLP) algorithms extract cardiovascular safety signals from clinical notes, while survival analysis models predict long-term outcomes [47]. For monoclonal antibodies and biologics, post-market RWE analysis can replace or reduce long-term animal toxicity studies by providing direct human outcome data on a large scale [6,11].

**Figure 3:**
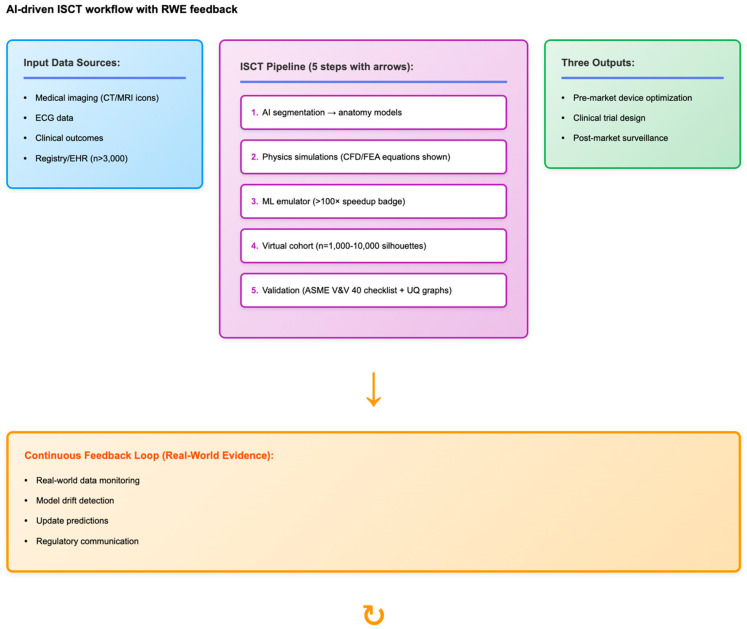
An AI-enabled in silico clinical trial (ISCT) workflow integrating real-world evidence (RWE) feedback for cardiovascular device evaluation. The left panel summarizes input data sources, including medical imaging, ECG data, and recording-based clinical outcomes. The central process illustrates five key steps: (1) AI segmentation to create anatomy models; (2) physics-based CFD/FEA simulations; (3) machine learning emulation for computational acceleration; (4) virtual cohort creation; and (5) model validation following ASME V&V 40 principles with uncertainty quantification. The right panel illustrates applications in pre-market device optimization, clinical trial design, and post-market surveillance. The feedback loop (bottom) emphasizes continuous RWE monitoring, drift detection, prediction updates, and regulatory communication, creating a closed-loop system that improves model accuracy over time. Blue denotes input data sources, purple denotes the ISCT computational pipeline, green denotes outputs, and orange denotes the continuous real-world evidence feedback loop. Straight arrows indicate sequential workflow steps, and circular arrows indicate iterative feedback loops based on real-world evidence.

Patient-specific simulations, when combined with virtual populations and validated with real-world data, enable in silico clinical trials that provide human-relevant predictions with minimal animal testing (Figure 3).

Digital twins, ISCTs, and RWE-driven surveillance provide a complementary triad to reduce animal use in the clinical translation and post-marketing phases. Currently, their role is primarily to focus on confirmatory animal and human studies, enhancing rather than replacing them entirely. Digital twins and ISCTs are constrained by computational cost, data requirements, and limited generalizability across centers, while RWE analyses are vulnerable to confounding factors, missing data, and coding biases in routine care. Therefore, careful definition of the context of use and rigorous external validation against independent registries are essential. Under these circumstances, AI tools in the clinical phase can enable strategic reduction in animal studies, first by replacing low-information design iterations and long-term exploratory toxicity studies, then by supporting increasingly more primary evidence as reliability increases.

## 5. Validation, Credibility, and Data Transparency

Regulatory acceptance depends on credibility evidence proportional to decision risk. The FDA’s CM&S guidance (2023) defines structured VVUQ requirements [14]. The framework includes:

1. QoI and CoU: Clearly define which decision the model is informing and at what stage of development/review it is.
2. Risk Assessment: Classify decision risk (low/moderate/high/critical) based on the potential impact of model error.
3. Verification: Ensure the model solves correctly (code testing, network convergence, numerical accuracy).
4. Validation: Demonstrate that the model represents reality for its intended use (external test data, clinical benchmarks).
5. UQ: Bound prediction with confidence intervals that account for parameter, model, and data uncertainty.
6. Applicability domain: Define the input ranges and boundary conditions within which model predictions are reliable.

For AI/ML models, transparency and calibration reflect the following expectations: data provenance, external validation on holdout sets, calibration curves, applicability domain definition, and performance monitoring should be clear [48,49] (Table 3).

Transparency practices (pre-registered modeling protocols, versioned code and data repositories, and Good Simulation Practices checklists) are increasingly emphasized for reproducibility [36,38]. Public availability of validation datasets and benchmarking problems accelerates model improvements and regulatory confidence across the community [10,36].

### Current Validation Gaps and Challenges

Despite progress, significant challenges remain:

- External validation gaps: Many AI cardiotoxicity models exhibit performance degradation on external datasets from different institutions or populations, highlighting overfitting and limited generalizability [29,49].
- Data heterogeneity: iPSC-CM analyses lack standardized protocols across laboratories, creating reproducibility challenges that complicate regulatory qualification [35,36].
- Regulatory uncertainty: While frameworks exist, specific acceptance criteria for AI-assisted NAMs in cardiovascular INDs continue to evolve, and case-by-case review still dominates [6,7].
- Computational accessibility: High-fidelity digital twins and large-scale ISCTs require significant computational resources, potentially limiting their adoption by smaller organizations [40,41].
- Mechanistic interpretability: Deep learning models often lack transparency in decision-making, leading to regulatory hesitancy for high-stakes decisions where mechanistic understanding is valuable [49].
- Species-specific gaps: Some cardiovascular mechanisms (e.g., complement activation, platelet-mediated thrombogenicity) are not adequately captured by current NAMs, necessitating the continued strategic use of animals in select applications [3,13].

Each category of AI-enabled NAM carries a distinct profile of strengths and weaknesses. In silico QSAR models and purely data-driven predictors are fast and inexpensive, making them attractive for early triage of thousands of compounds. but their performance collapses when training data is sparse, biased, or mechanistically incomplete [28,29]. Human in vitro systems such as iPSC-CMs and MPS provide richer biology and can reveal human-specific failure modes, but their variability, inter-laboratory protocol differences, and limited systemic context make robust validation difficult [34,35]. High-fidelity digital twins and ISCTs capture multi-scale physiology but require extensive imaging, clinical data, and computational resources, which limits widespread application and may introduce selection bias toward well-characterized populations [40,41]. A pragmatic way forward is a hybrid validation strategy: computational models narrow the design space, human in vitro systems interrogate underlying mechanisms, and strategically selected animal or clinical studies provide the final cross-check in contexts where alternatives are lacking [38,46]. Validation frameworks, such as ASME V&V 40 and the FDA’s new AI/ML lifecycle guide, are most effective when used to organize this community, rather than universally certifying any NAM as validated.

## 6. Ethical and Economic Outlook

AI-powered NAMs address the 3Rs while simultaneously reducing costs and accelerating cardiovascular development processes. Monoclonal antibody therapies for cardiovascular indications (such as PCSK9 inhibitors (evolocumab, alirocumab) and anti-inflammatory agents (such as canakinumab, which targets IL-1β for atherosclerosis)) typically require 6-month non-human primate toxicology studies estimated to cost $2 million or more [11,12]. NAM-based PBPK/QSP modeling, AI-assisted cardiotoxicity assessment, and human microphysiological systems could eliminate 50–70% of these exploratory studies in well-defined context of use, both lowering costs and improving human relevance [6,11,12]. However, these projections are highly dependent on the maturity of individual methods, the upfront investments required, and the regulatory acceptance achieved in specific applications.

### 6.1. Economic Impact and Return on Investment

High-level projections suggest that successfully implemented NAMs can reduce animal use and shorten development times [5,6,7,11,12], but these benefits are not uniform across product types or organizations. From an industry perspective, economic evaluation must balance three main components:

1. Capital expenditures (CapEx): Investments in microphysiological platforms, imaging and automation infrastructure, high-performance computing, and data engineering teams. For example, establishing an in-house microphysiological system and AI analysis pipeline can require seven-figure investments in equipment, software, and specialized personnel, especially for large biopharmaceutical programs.
2. Operating expenses (OpEx): Ongoing costs for analysis consumables, cloud or on-premises computing, data storage, maintenance, and quality assurance. While unit costs for in vitro assays are lower than for large-scale animal studies, early implementation often requires redundancy (running both traditional studies and NAMs in parallel) during the learning curve.
3. Downstream savings and risk reduction: Fewer large animal studies, shorter timelines for lead optimization and dose selection, reduced likelihood of late-stage failure, and the ability to reuse validated platforms across projects. These benefits are most pronounced for primate-intensive modalities (e.g., monoclonal antibodies and certain biologics) and device programs where in silico design can replace multiple iterative animal studies [6,11,12,32,33].

Conceptually, a positive return on investment is most likely when:

- Baseline animal burden and cost per program are high (e.g., primate-intensive biologics, complex implantable devices);
- NAMs can be reused across multiple assets, indications, or device generations;
- Regulators can consider NAM data as primary or co-primary evidence for specific areas of interest, resulting in a true reduction in animal testing rather than simply adding new assays.

For small molecules with relatively inexpensive preclinical packages, the business model may be based on improving human predictive value (avoiding expensive late-stage failures) rather than direct animal cost savings. For smaller companies, partnering with contract research organizations or shared NAM platforms may be more cost-effective than building full internal capacity. In general, current cost estimates (50–70% reduction in selected animal studies) should be interpreted as scenario-based projections rather than guaranteed averages; they are highly dependent on the application strategy and regulatory acceptance in each context of use.

### 6.2. Practical Barriers to Widespread Implementation

Despite promising pilot studies, several practical barriers hinder the widespread adoption of AI-assisted NAMs in cardiovascular R&D:

- Technology availability and scalability: Advanced organ-on-chip platforms, human iPSC-CMs, and high-fidelity digital twin simulations are currently concentrated in a small number of specialized suppliers and academic centers. Procurement costs, long lead times, and limited production capacity may hinder routine use, especially for smaller companies and public laboratories.
- Standardization and assay robustness: The lack of harmonized protocols, reference materials, and interlaboratory proficiency testing makes it difficult to compare results across centers. Even for seemingly mature systems such as hiPSC-CM assays, differences in cell source, culture conditions, and readout methods can lead to different results [34,35,36]. Standardization efforts by the OECD, FDA, and EMA are ongoing but incomplete [8,9,36].
- Workforce and training constraints: Effectively deploying AI-powered NAMs requires interdisciplinary teams combining regulatory science, toxicology, bioengineering, data science, and software engineering. Many organizations, especially small and medium-sized businesses, struggle to recruit or train staff with this skill set. Regulators also need the time and resources to build in-house expertise in evaluating complex AI and simulation-based applications.
- Regulatory and institutional inertia: Even if alternative methods demonstrate strong performance, entrenched standard operating procedures, contracts with animal shelters, and risk-averse corporate cultures can slow change. Sponsors may be hesitant to trust NAMs for important decisions if they are unsure how regulators will evaluate the evidence.
- Access inequities: Larger multinationals are more likely to afford state-of-the-art NAM platforms and participate in regulatory pilot programs, while smaller organizations risk being left behind. If this is not addressed, a two-tiered ecosystem can emerge in which only well-resourced actors benefit from NAM-driven efficiencies and ethical gains.

Addressing these barriers will require coordinated investment in shared infrastructure (e.g., public NAM platforms, reference datasets), expanded education initiatives for both regulators and sponsors, and guidance that explicitly recognizes scalable options for smaller organizations.

### 6.3. Ethical Considerations and Algorithmic Bias

Standardized human assays and computational models can reduce animal numbers and improve reproducibility by eliminating interspecies variability. However, the transition to AI-assisted NAMs raises new ethical challenges that extend beyond animal welfare. Regulators currently emphasize that limited, strategically designed confirmatory animal or human studies should continue until NAM validation is comprehensive across relevant safety endpoints [5,6]. The transition is explicitly framed as a “strategic reduction” not immediate elimination, with animals remaining necessary for:

- Mechanisms for which human biology is not fully understood;
- Systemic toxicity endpoints not yet modeled in vitro/in silico;
- Immunogenicity assessment for some biologics;
- Long-term durability and biocompatibility for implantable devices.

Furthermore, AI-powered NAMs may unintentionally encode and reinforce bias. Models trained on clinical datasets that underrepresent certain age groups, ethnicities, or comorbidities may underperform for these very populations, leading to unequal safety margins or missed risk signals. For example, if cardiotoxicity prediction models are trained primarily on data from high-income settings, their predictions may be less reliable for patients exposed to different background treatments or environmental risk factors.

Therefore, ethical implementation requires:

- Documentation of the composition of the training data and known gaps;
- Evaluation of performance across clinically relevant subgroups;
- Transparency about uncertainty and limitations when applying models outside of development contexts;
- Governance mechanisms (such as independent auditing, stakeholder oversight, and post-market monitoring) to detect and respond to systematic errors.

From a broader ethical perspective, NAM adoption should be evaluated not only in terms of the number of animals protected, but also in terms of who benefits and who is exposed to residual risk. A people-centered NAM strategy balances animal welfare gains with commitments to equity, transparency, and accountability in AI-powered decision-making.

**Figure 4:**
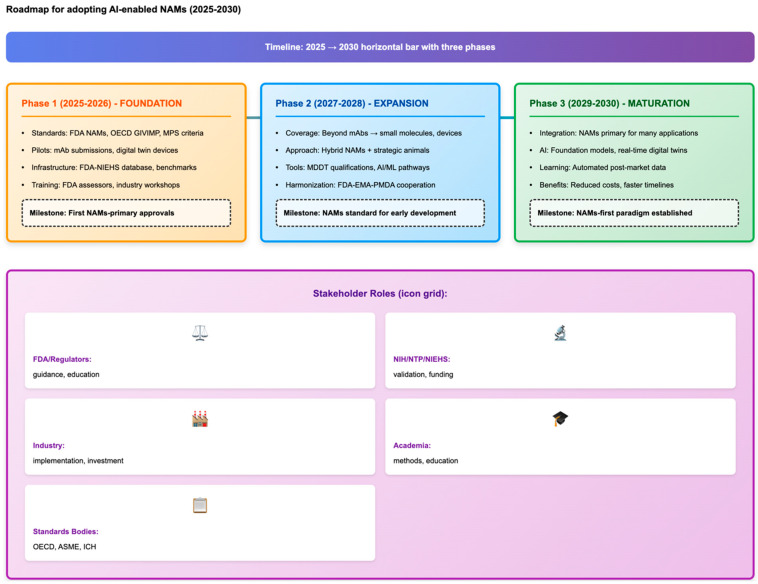
Roadmap for the adoption of AI-powered NAMs (2025–2030). A three-phase implementation timeline to achieve the FDA’s goal of making animal testing the exception, not the rule, in cardiovascular development. **Phase 1 (2025–2026, Foundation)**: Standards and guidelines are established (implementation of FDA NAMs, OECD GIVIMP, microphysiological system performance criteria), pilot programs are launched (mAb NAM applications, digital twin devices), data infrastructure is established (FDA-NIEHS toxicity database, benchmark datasets), and training is provided (FDA assessor capacity, industry workshops). Key milestone: First NAMs-primary IND/IDE approvals. **Phase 2 (2027–2028, Expansion)**: Expand scope beyond mAbs to small molecules and device categories, implement hybrid NAMs + strategic animal approaches, develop regulatory tools (MDDT qualifications, AI/ML pathways), and ensure international harmonization (FDA-EMA-PMDA mutual recognition). Key milestone: NAMs become standard practice for early cardiovascular development. **Phase 3 (2029–2030, Maturation)**: Integrate with NAMs as primary proof of concept for many applications, leverage advanced AI systems (multimodal foundation models, real-time digital twins), apply automated continuous learning from post-market data, and realize economic benefits. Key milestone: Establish NAMs as a priority paradigm with strategic animal use for critical gaps. Stakeholder roles: FDA/Regulators (guidance, education, pilots), NIH/NTP/NIEHS (validation, datasets, funding), Industry (implementation, applications, investment), Academia (methods, validation, education), and Standards Bodies (OECD, ASME, ICH compliance). Orange, blue, and green panels represent Phase 1 (Foundation), Phase 2 (Expansion), and Phase 3 (Maturation), respectively; the purple section denotes stakeholder roles across phases.

As a synthesis of the ethical, economic, and operational considerations discussed above, Figure 4 outlines a practical 2025–2030 roadmap demonstrating how AI-enabled NAMs can progressively support the strategic reduction in animal studies in cardiovascular development and identify key milestones required for broader regulatory and industrial adoption.

## 7. Limitation and Future Directions (2025–2030)

Despite their promise, several technical and regulatory limitations constrain the near term of animal studies. A key limitation is the lack of standardized, high-quality, FAIR (Findable, Accessible, Interoperable, Reusable) data for training and validating AI models. Publicly available toxicity databases often exhibit heterogeneity in endpoints, experimental protocols, and data curation and may contain conflicting results [49]. Most existing NAMs also capture only certain aspects of cardiovascular biology: Peripheral vasculature, immune responses, and multi-organ interactions are underrepresented in many iPSC-CM and organ-on-chip models, while most cardiac digital twins still focus on electrical and mechanical function rather than systemic inflammation or thrombosis [50,51]. Addressing these gaps will require coordinated investment in benchmark datasets, multi-organ microphysiological systems, and integrated modeling frameworks. A second priority is cross-institutional and multi-center validation. Ongoing FDA-NIEHS-NTP collaborations aim to create benchmark datasets for NAM and AI model validation and complete the NIH-FDA Memorandum of Understanding (2025) on the evaluation of cardiac microphysiological systems and digital twins [51]. These initiatives aim to create open-access repositories of validated cardiovascular toxicity data, define standardized performance criteria, and coordinate multicenter validation studies to assess reproducibility across laboratories. Align expectations across CBER, CDER, and CDRH will be crucial to avoid fragmented requirements and encourage sponsors to invest in collaborative validation studies.

Data access and privacy concerns are driving interest in unified, privacy-preserving AI. Training models on distributed EHR and registry data without centralizing patient-level information can increase generalizability and reduce selection bias while maintaining compliance with data protection regulations [52]. However, harmonizing data schemas, managing communication overhead, and establishing governance structures for interagency collaborations remain open challenges. On the regulatory side, hybrid “regulatory trial environments” are emerging as a practical mechanism to mitigate innovation risk. Pilot programs that allow for the iterative delivery of NAM packages with real-time feedback from regulators on reliability evidence and the context of use can help sponsors refine their models before formal review [6,7]. Such trial environments transform NAM assessment from a one-time pass/fail decision into a shared learning process between regulators and developers, accelerating both parties’ understanding of what constitutes sufficient evidence.

Standardized reporting and transparency practices will determine whether individual NAM success stories can be generalized. Guidelines such as the OECD GIVIMP and FDA NAM implementation documents emphasize traceable data lineage, uncertainty documentation, and clear definition of model assumptions [8,36]. Adopting structured reporting templates similar to CONSORT for clinical trials or TRIPOD for predictive models will facilitate meta-analyses and systematic reviews, making it easier for regulators to assess the full body of evidence. Finally, the rapid evolution of advanced AI architectures (multimodal transducers, broad language models, and core models for imaging and signaling) presents both opportunities and new risks [53]. These models can integrate heterogeneous data streams (omics, imaging, waveforms, clinical text) to support richer cardiovascular NAMs, but they also raise questions about explainability, robustness to dataset variation, and the potential for emerging failure modes. Future studies should prioritize rigorous external validation, transparency about model limitations, and close collaboration between AI developers, clinicians, and regulators.

Overall, the 2025–2030 timeframe should not be considered a guaranteed deadline for animal replacements, but rather a critical window for establishing the infrastructure, evidence base, and governance mechanisms needed to make animal testing the exception rather than the default. Progress will depend on sustained investment in shared resources, clear regulatory incentives, and inclusive participation by academia, industry (large and small), regulators, and patient and animal welfare stakeholders.

## 8. Conclusions

AI-powered alternative methods are beginning to reshape cardiovascular translational research by not completely replacing animal studies but rather enabling a more strategic and evidence-driven use. Regulatory modernization, including the FDA’s 2025 Roadmap and complementary EMA and OECD frameworks, provides a clearer structure for integrating NAMs across the development pipeline. The cardiovascular field is particularly well-positioned to benefit due to the long-recognized animal–human discordance in key safety areas and the increasing availability of human-relevant data streams, from iPSC-derived systems to large, real-world clinical datasets.

However, realizing this opportunity requires acknowledging current limitations. Many AI-powered models are context-dependent, susceptible to dataset variation, and have undergone variable validation across institutions. Microphysiological systems and digital models often lack standardized protocols, and regulatory acceptance still depends on transparent, reproducible evidence generated across multiple centers. Consequently, NAMs currently function best as complementary tools for screening, prioritization, and mechanistic interpretation. However, focused animal studies are still necessary for remaining uncertainties that cannot yet be modeled in vitro or in silico.

Therefore, the next few years should not be understood as a deadline for a renewal, but rather as a critical timeframe for establishing the necessary validation infrastructure for strategic mitigation. Multicenter studies, harmonized VVUQ frameworks, assessor training, and shared benchmark datasets will be central to ensuring reliability. Early adopters who demonstrate successful regulatory submissions using NAM-supported evidence will help set precedents and increase confidence in the field. Through coordinated investment and careful validation, cardiovascular innovators can transition to a development paradigm where NAMs handle the majority of routine evidence generation and animal studies are used only when necessary. This trajectory supports not only ethical imperatives but also enhanced scientific credibility and more efficient development programs (Box 1).

Box 1 **Glossary of Key Terms.**

- New Approach Methodologies (NAMs): Alternative methods to animal testing, including in vitro systems, computational models, and human-derived data.
- ASME V&V 40: The American Society of Mechanical Engineers’ standard for verification and validation of computational models.
- Context of Use (CoU): The specific regulatory purpose and scope for which a model or method qualifies.
- Drug-induced QT prolongation: A condition in which certain medications cause the heart’s electrical charging to take longer than normal.
- İn silico Clinical Trial (ISCT): Computational simulation of patient cohorts to predict intervention outcomes.
- Physiologically Based Pharmacokinetics (PBPK): Mechanistic modeling of drug distribution based on organ physiology.
- MPS (Microphysiological Systems): Organ-on-a-chip platforms that recapitulate human tissue function.
- iPSC-CM: Induced pluripotent stem cell-derived cardiomyocytes.
- Uncertainty Quantification (UQ): Statistical characterization of model prediction uncertainty.
- hERG: Human ether-à-go-go-related gene; Potassium channel target for cardiotoxicity screening.
- Question of Interest (QoI): The specific question a computational model is designed to answer.

## Figures and Tables

**Table 1:** Comparative regulatory requirements across jurisdictions.

Aspect	FDA (US)	NMPA (China)	PMDA (Japan)	MHRA (UK)	EMA (EU)
Alternative method acceptance	Medium-High	Low-Medium	Medium	High	Medium
Computational modeling pathway	MDDT program	Case-by-case	Sōdan consultation	ILAP	Innovation Task Force
Primary evidence acceptance	Yes (with validation)	Rare	Rare	Yes	Variable by member state
Timeline for new methods	6–12 months	18–24 months	12–18 months	4–10 months	12–18 months
International data acceptance	Yes	Domestic required	Yes, with supplement	Yes	Yes, within the EU
ASME V&V 40 approval	Recognized standard	Under evaluation	Recognized	Recognized	Recognized

Note: MDDT = Medical Device Development Toolkit; ILAP = Innovative Licensing and Access Pathway; ASME V&V 40 = American Society of Mechanical Engineers Verification and Validation Standard 40. Timeline estimates represent typical review times for new alternative method applications through 2025. Acceptance levels and pathways continue to evolve; developers should confirm current requirements with relevant regulatory agencies.

**Table 2:** Representative AI models supporting NAMs in cardiovascular research (2019–2025).

Use Case	Representative Model	Key Output	Implication for Animal Use	Role Reference
hERG risk prediction	DeepHIT/GBDT models	Block probability	Filters compounds pre-in vivo; reduces exploratory animal studies by 60–80%	[28,29]
QT prolongation (clinical)	QTNet CNN in ECGs	ECG-based QT risk	Guides safer dosing; informs trial design; reduces confirmatory studies	[33]
iPSC-CM phenotyping	CNN in high-content imaging	Toxicity morphology scoring	Replaces exploratory animal safety screenings with human testing	[35]
Mechanistic + ML surrogate	Cardiac electrophysiology emulator	Rapid population UQ	Virtual population modeling replaces animal variability studies	[37]
Hybrid exposure-response	PBPK/QSP + ML-augmented parameterization	Organ/tissue exposure and safety margin, dose optimization	Reduces cross-species extrapolation; accelerates mAb development	[11,12]
Network pharmacology	Graph neural networks	Pathway toxicity links	Generates mechanistic hypotheses without animals	[39]

**Table 3:** Credibility evidence mapping for cardiovascular AI/NAMs.

Application	Decision Risk	Basic Credibility Tasks	Reference Framework
Early compound triage (hERG AI)	Low	External test set validation; calibration curves; applicability domain documentation	[10,49]
iPSC-CM/MPS analyses	Moderate	Interlaboratory reproducibility; orthogonal endpoint validation; performance criteria	[8,36]
Device design iteration (CFD/FEA)	Moderate-High	Code validation; validation against benchtop and clinical data; sensitivity analysis; UQ	[14,15]
Regulatory labeling (primary evidence)	High-Critical	Full VVUQ per CoU; validation against independent clinical datasets; comprehensive sensitivity analysis; bias assessment	[14,15]

## Data Availability

All supporting data is included in the article.
